# Pain inhibition—the unintended benefit of electrically elicited muscle strengthening contractions

**DOI:** 10.1186/s12891-023-06243-x

**Published:** 2023-02-18

**Authors:** Katherine S. Rudolph, Matthew Cloutier, Scott Stackhouse

**Affiliations:** 1 Department of Physical Therapy, University of New England, 716 Stevens Ave., Portland, ME 04103 USA; 2 College of Osteopathic Medicine, University of New England, 11 Hills Beach Road, Biddeford, ME 04005 USA

**Keywords:** Neuromuscular electrical stimulation, Pain inhibition, Conditioned pain modulation, Quadriceps, Neuromuscular inhibition, Muscle inhibition

## Abstract

**Background:**

Neuromuscular electrical stimulation (NMES) is effective in muscle strengthening after orthopedic injury particularly when muscle activation failure is present, but the associated pain can be a barrier. Pain itself can produce a pain inhibitory response called Conditioned Pain Modulation (CPM). CPM is often used in research studies to assess the state of the pain processing system. However, the inhibitory response of CPM could make NMES more tolerable to patients and could improve functional outcomes in people with pain. This study compares the pain-inhibitory effect of NMES compared to volitional contractions and noxious electrical stimulation (NxES).

**Methods:**

Healthy participants, 18–30 years of age experienced 3 conditions: 10 NMES contractions, 10 bursts of NxES on the patella, and 10 volitional contractions on the right knee. Pressure pain thresholds (PPT) were measured before and after each condition in both knees and the middle finger. Pain was reported on an 11-point VAS. Repeated measures ANOVAs with 2 factors: site and time were performed for each condition followed by post-hoc paired t-tests, with Bonferroni correction.

**Results:**

Pain ratings were higher in the NxES condition compared to NMES (*p* = .000). No differences in PPTs prior to each condition were observed but PPTs were significantly higher in the right and left knees after the NMES contractions (*p* = .000, *p* = .013, respectively) and after the NxES (*p* = .006, *P*-.006, respectively). Pain during NMES and NxES did not correlate with pain inhibition (*p* > .05). Self-reported pain sensitivity correlated with pain during NxES.

**Conclusion:**

NxES and NMES produced higher PPTs in both knees but not in the finger, suggesting that the mechanisms responsible for the reduction in pain are located in the spinal cord and local tissues. Pain reduction was elicited during the NxES and NMES conditions regardless of the self-reported pain ratings. When NMES is used for muscle strengthening significant pain reduction can also occur, which is an unintended benefit of the intervention that could improve functional outcomes in patients.

## Background

Muscle weakness is ubiquitous following musculoskeletal injury or surgery such as anterior cruciate ligament injury or total knee arthroplasty. Rehabilitation to restore the force producing capacity of a muscle can be compromised by the inability to fully activate a muscle voluntarily [1, 2]. One mechanism of reduced voluntary muscle activation is arthrogenous muscle inhibition, which has been related to joint distension associated with effusion [1, 2] and nociceptive signaling [3, 4]. A number of treatment interventions are effective in managing arthrogenous muscle inhibition. EMG biofeedback is a mechanism to make electrical signals generated during muscle contraction visible to patients. Patients can be trained to increase the EMG level by increasing volitional motor unit recruitment and/or rate coding that can increase force production. Biofeedback has been shown to be effective for increasing force production in numerous populations [5–7]. Another treatment approach involves neuromuscular electrical stimulation (NMES) [8, 9] which bypasses the central nervous system and activates muscle directly through the activation of motor neurons. Moreover, NMES increases the excitability of the nervous system at the spinal and cortical levels [10–12], which can help to overcome muscle inhibition thus enabling volitional muscle contractions to be more effective. NMES is beneficial for people with or without arthrogenic muscle inhibition because of the manner in which motor units are recruited. Volitional recruitment follows Henneman’s Size Principle, thus motor units are recruited from small to large size [13, 14]. Large, strong motor units are typically only recruited volitionally during high force or rapid contractions and may not be activated during typical strengthening programs. NMES activates motor axons so recruitment is dictated by the biophysics of the axon that is largely related to neuron’s diameter [11]. Therefore, large diameter neurons may be activated before smaller diameter neurons [15], although the recruitment order is not reversed in the strict sense because proximity to the stimulation electrodes also plays a role [15, 16]. Nonetheless, NMES activates more motor units capable of generating large forces than volitional contractions at the same force level making strengthening more effective.

NMES will also activate neurons that transmit noxious stimuli that can lead to significant discomfort, which is a potential barrier to its use [17, 18]. However, the benefits of NMES outweigh the discomfort so patient education is key to its success. One aspect of NMES that is largely overlooked is the potential for pain inhibitory mechanisms to be activated via the noxious sensations of the stimulation. Conditioned pain modulation (CPM) is a well-documented pain-relieving phenomenon that is described as “pain inhibiting pain”, which is called diffuse noxious inhibitory control in animals [19]. This type of pain inhibition is measured by testing pain thresholds before and after a painful conditioning stimulus is applied. Thus, any painful conditioning stimulus has the potential to elicit this type of pain inhibition in people with typical nervous systems. While the most common reason to use NMES in musculoskeletal conditions is to increase the force generating capacity of a muscle, the pain sensations of the contractions could also serve as conditioning stimuli that inhibits pain, which would be an unintended benefit of NMES, but this has not been investigated experimentally. The purpose of this study was to apply NMES using a protocol similar to that used for muscle strengthening, and explore the pain inhibition. We compared the effects of NMES-elicited contractions to voluntary muscle contractions and to that of noxious electrical stimulation (NxES) without muscle contraction to control for the independent effects that pain and exercise can have on pain inhibition. We hypothesized that electrical stimulation of muscle that would be painful thus would produce similar levels of pain inhibition as noxious electrical stimulation. We also hypothesized that, when the painful stimuli were applied to the right leg, pain inhibition would be present in both knees, and in the finger because mechanisms involved in CPM have been identified at the level of the spinal cord as well as higher brain centers. Healthy young adults were tested so that impaired pain processing, which is often associated with musculoskeletal pain, would not influence the results.

## Methods

Participants in this exploratory study were recruited from 9/1/2018 thru 8/31/2019 from a sample of convenience of young healthy men and women between the ages of 18–30 who gave informed consent that was approved by the Institutional Review Board of the University of New England (#18.09.05–003). The research protocol was registered on ClinicalTrials.gov on 15–03-2022 (Identifier: NCT05280522). The rights of all participants were protected and each participant was informed that their data would be submitted for publication. Exclusion criteria included: uncontrolled high blood pressure, diabetes, leg or spine injury in the past 12 months that required the care of a medical practitioner, pain in the previous 6 months lasting more than 3 days, fibromyalgia or other chronic pain condition, neurological condition, dizziness, or unexplained falls.

**Fig. 1:**
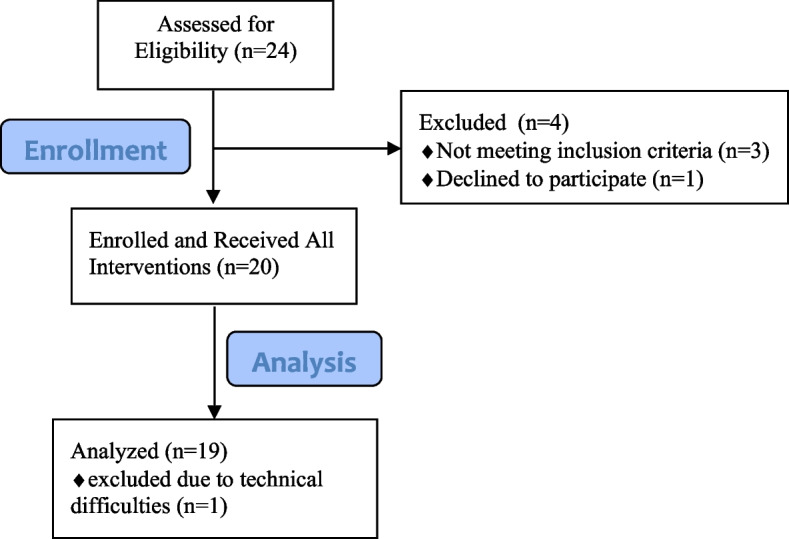
Study Flow Diagram

Participants that were enrolled included 19 young healthy individuals aged 19–30 ($\overline{x }$=24.4, ± 2.5) years; 9 were female (Fig. 1).

Testing took place over two sessions, in the Motion Analysis Laboratory of the University of New England separated by at least one hour. In session 1, participants were familiarized with any procedures that could cause pain, and the maximum voluntary isometric contraction (MVIC) of the quadriceps muscles was measured.

In session 2, participants took part in three experimental conditions: Volitional contractions at 20% MVIC, NMES contractions at 20% MVIC and noxious stimulation at the highest tolerable level. The NMES condition was applied last because it can cause muscle fatigue that would interfere with the volitional condition. The order of the volitional contractions and noxious stimulation conditions was alternated between first and second in testing order for consecutive participants.

Participants were positioned in an isokinetic dynamometer with the knee flexed to ~ 90°. After 3–4 submaximal warm up contractions, participants generated maximum knee extension force while vigorous verbal encouragement was provided. Two trials were collected with ~ 30 s between trials. The maximum forces from the 2 trials were averaged and used to determine the target force of 20% MVIC.

### Volitional contractions (VOL)

Participants were provided visual feedback showing the 20% MVIC force and instructed to produce the target force for 10 s then relax for 50 s. The timing was provided by the research personnel. This was repeated 10 times.

### Electrically elicited contractions

Two, large, self-adhesive electrodes (~ 8 × 12 cm) were applied to the proximal and distal thigh. An electrical stimulation unit (EMPI PV300, DJO Global, Vista, CA) delivered monophasic square wave pulses (duration 400 μs, frequency 40 Hz) for up to 30 s. The amplitude of the current was slowly increased to generate 20% MVIC force and the amplitude was recorded. The timing used during neuromuscular stimulation for strengthening is 10 s on: 50 s off for 10 contractions. Ten second contractions allows time for sufficient overload to the muscle to elicit strength gains and 50 s off time provides sufficient time for the muscle to recover physiologically. Ten contractions, 10 s in duration separated by 50 s rest periods were generated.

### Noxious Stimulation (NxES)

A monopolar electrode setup was used to deliver the noxious stimulation. A 1 × 2 cm self-adhesive electrode placed over the base of the patella. A large electrode served as the dispersive electrode that was positioned over the distal quadriceps muscles. Monophasic square wave pulses (duration 400 μs, frequency 80 Hz) for up to 30 s while the amplitude was slowly increased to the maximum tolerated by the subject. The maximum tolerated current amplitude was recorded and used during the NxES condition. Noxious stimulation for pain relief is typically delivered 10 s on: 10 s off for 15–20 min 6. However, we maintained similar timing for all of the conditions to allow comparison. Thus, the volitional contractions, electrically elicited contractions, and noxious stimulation were all performed for 10 s on: 50 s off.

Participants also rated the pain during each of the 10 s muscle contractions and the application of NxES on a 100-point visual analog scale. The pain ratings were averaged across all 10 stimuli or contractions and the average was used in the analyses.

### Pressure pain threshold testing

Pressure pain threshold (PPT) was used to assess pain as described by [20], using a pressure algometer (Algomed, Medco, Durham, NC). Pressure was applied over the quadriceps tendon 1 cm proximal to the patella through a 1.0 cm diameter tip at a rate of 30 kPa/s [20]. The subject was instructed, using a standard script, to indicate when the sensation changed from deep pressure to pain. While the muscle contractions and noxious stimulation were performed on the right quadriceps muscles or tendon, respectively, pressure pain thresholds were measured in the same knee, as well as the contralateral knee (the same level of the spinal cord) and in the distal phalanx of the middle finger of the right hand, to assess pain inhibition at a different spinal level. Three consecutive PPTs were measured at each site, ≥ 30 s between each measurement and the last 2 measurements were averaged and used in the analysis. The difference between the pre- and post-condition PPTs was operationally defined as conditioned pain modulation (CPM) and used in the analysis.

A subset of 10 participants completed the Pain Sensitivity Questionnaire (PSQ) to assess general sensitivity to pain. The PSQ is a 17-item self-report questionnaire that involves rating pain in imagined painful situations that includes different types of pain and different body locations [21, 22]. In healthy subjects, The PSQ score relates to pain intensity ratings but not pain thresholds [21].

### Statistical analysis

To determine whether PPT measurements were different at the three body sites, a repeated measures ANOVA with 2 factors: intervention (levels: volitional contractions, electrically elicited contractions, noxious stimulation) and site (Levels: RKnee, LKnee, Finger) was run on the pre-intervention PPT measurements because they would have been unaffected by the interventions. To assess our a-priori hypothesis that noxious stimulation and electrically elicited contractions would produce a pain inhibition while volitional contractions would not, we performed a repeated measures ANOVA with 2 factors: site (RKnee, LKnee, Finger) and time (pre, post) for each condition followed by post-hoc paired t-tests, with Bonferroni correction for multiple comparisons. To determine if there was a significant difference in pain inhibition induced by the electrical contractions and the noxious stimulation, repeated measures ANOVA with 2 factors: Intervention (electrical contractions, noxious stimulation) and time (pre, post) were performed separately on data measured at the RKnee, LKnee and Finger.

## Results

**Fig. 2:**
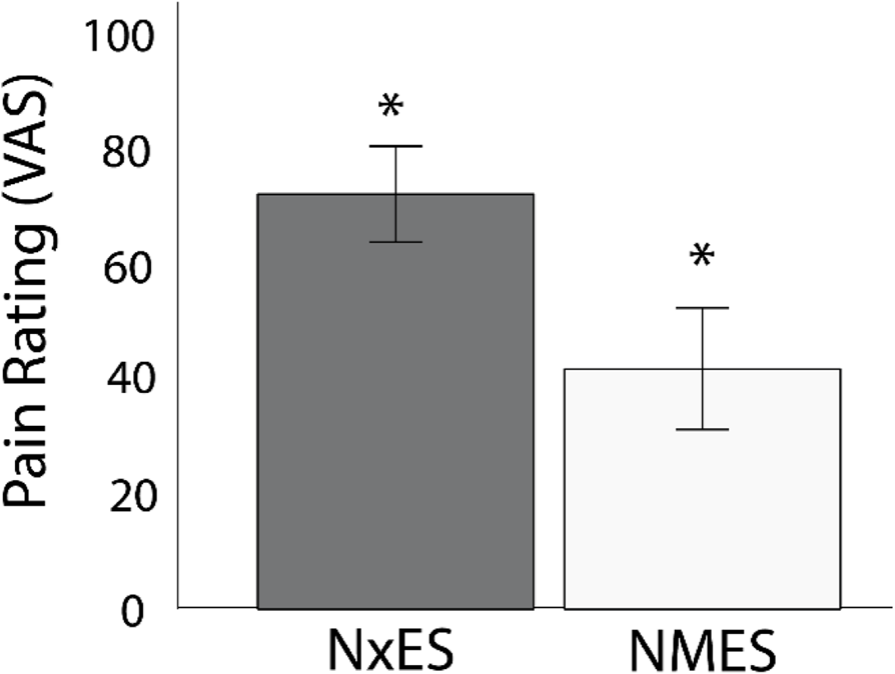
Average pain ratings during the NMES contractions and NxES. Error bars represent 95% CI. * indicates a statistically significant difference at the level *p* ≤ .05

The target force for the volitional and electrically elicited contractions was 20% MVIC, and the average, measured force during the 10 contractions was greater during the volitional contractions (28.7%MVIC (± 2.4)) than during the electrically elicited contractions (23.7%MVIC (± 5.7); t = 4.489, *p* = 0.000). Pain ratings during the electrical muscle contractions were lower than in the noxious stimulation as illustrated in Fig. 2 (t = -5.859, *p* = 0.000). None of the participants experienced any harm or unintended effects of the study procedures. Nor did any of the participants experience discomfort during the volitional contractions.

### Differences between body sites

In all figures, an increase in the post-intervention PPT indicates pain inhibition. There was a main effect of body site (F = 23.995, *p* = 0.000, partial eta squared = 0.575) and post-hoc tests showed that prior to each condition, PPTs measured at the finger were lower than those at either the RKnee (*p* = 0.000) and LKnee (*p* = 0.004), but those measured from the RKnee and LKnee were no different (*p* = 0.337). The lack of difference in pre-condition PPTs illustrates that a sufficient wash-out period was provided between conditions.

### Differences between painful interventions

**Fig. 3:**
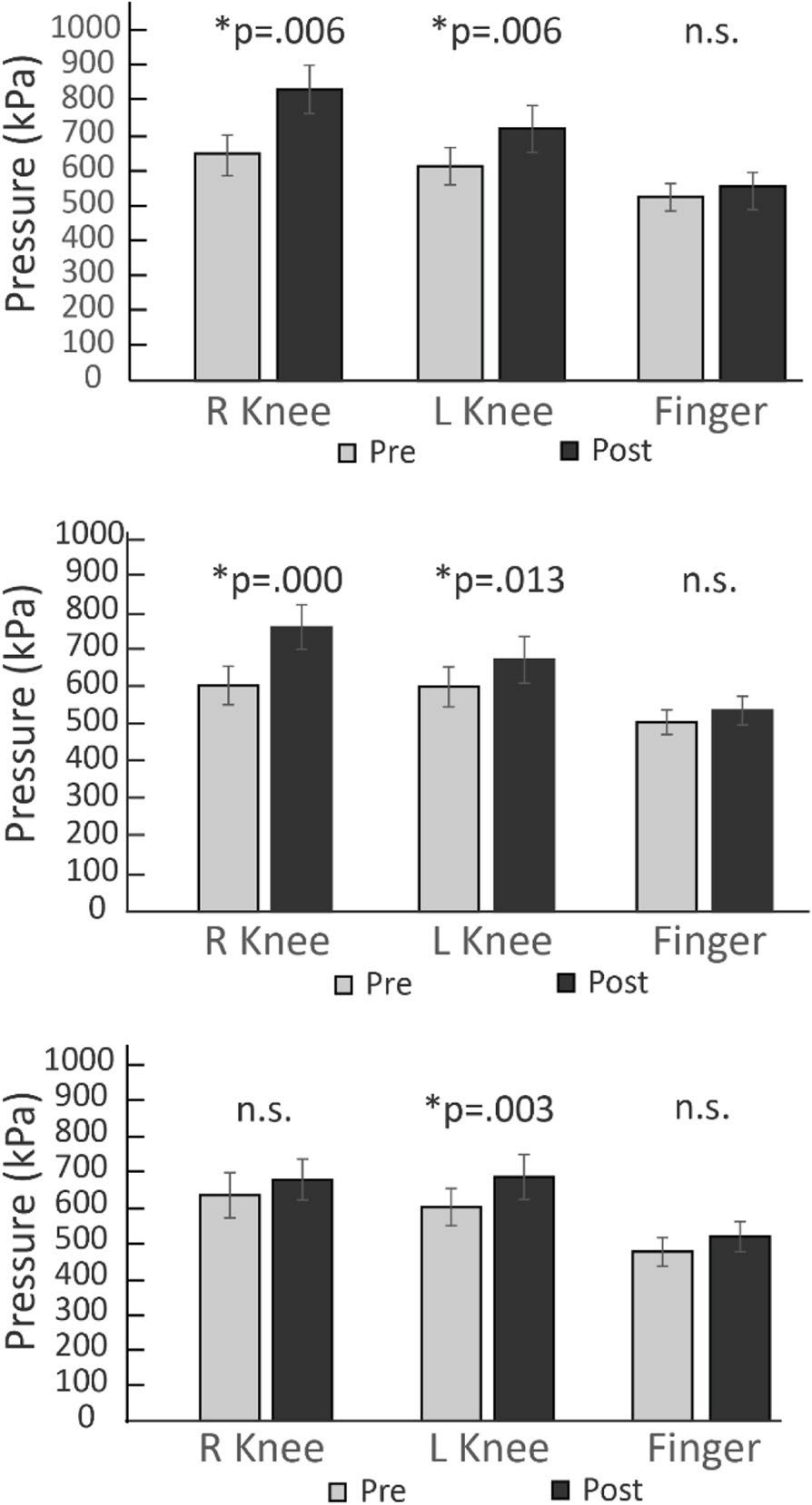
Pressure pain thresholds (PPT) before (gray) and after (black) NxES (top), NMES (middle) and Volitional (bottom) contraction in the right treatment knee, contralateral left leg, and the right finger. Error bars represent 95% CI. * indicates statistical significance at the level *p* ≤ .05

In response to the volitional contractions performed on the right side, only the left knee showed a significant pain inhibitory response (Fig. 3, bottom). After the electrically elicited contractions were generated on the right side, pain inhibition was observed at the RKnee and LKnee, but not at the finger (Fig. 3, middle). In response to noxious stimulation performed on the right knee, pain inhibition was observed in both RKnee and LKnee, but not at the finger (Fig. 3, top). Noxious condition (top) showed inhibition in the treatment (*p* = 0.006) and contralateral knees (*p* = 0.006). As shown in Fig. 2, NMES contractions (middle) showed pain inhibition in the treatment limb (*p* = 0.000) and the contralateral knee (*p* = 0.013), while volitional contractions showed inhibition only in the contralateral knee (*p* = 0.003).

Differences in PPT before and after the painful conditions can be seen in see Table 1.

**Table 1:** Changes in PPT after the NMES and NxES at the different body sites

		**Electrical contractions**	**Noxious stimulation**	**Main and Interaction Effects (F, *p*-value, eta squared)**
RKnee	Pre Post	603.8 (224.0) 759.5 (259.1)	649.8 (229.0) 833.7 (300.0)	Intervention: F = 5.159, *p* = .036*, partial eta squared = .223 Time: F = 45.562, *p* = .000*, partial eta squared = .717 Time x Intervention: F = 1.206, *p* = .287
LKnee	Pre Post	600.4 (232.8) 672.2 (270.5)	613.1 (234.0) 721.9 (287.0)	Time: F = 12.881, *p* = .002*, partial eta squared = .417 Intervention: F = 1.149, *p* = .298 Time x Intervention: F = 1.035, *p* = .323

*PPT* pressure pain threshold, *NMES* Neuromuscular electrical stimulation, *NxES* Noxious electrical stimulation

^*^indicates statistical significance at the level *p* ≤ .05

In the Right knee, there were main effects of intervention and time, but no interaction effects were observed. On the left side, there was a main effect of time only.

### Relationships of pain inhibition with muscle force and pain perception

To explore relationships between muscle contraction force and pain inhibition, and between pain perception and pain inhibition, Pearson Product Moment correlations were calculated between the variables at the three testing sites. No significant correlations were observed between the force of the muscle contractions and the change in pressure pain thresholds (Table 2).

**Table 2:** Relationships between ΔPPT and force of VOL and the NMES contractions

Pairs	Pearsons r, *p* value
VOL F vs. ΔPPT R Knee	-.094, *p* = .702
VOL F vs. ΔPPT L Knee	.076, *p* = .756
VOL F vs. ΔPPT Finger	-.071, *p* = .772
NMES F vs. ΔPPT R Knee	.361, *p* = .129
NMES F vs. ΔPPT L Knee	-.091, *p* = .710
NMES F vs. ΔPPT Finger	.105, *p* = .669

*F* Force, *NMES* neuromuscular electrical stimulation, Δ*PPT* change in pressure pain threshold, *VOL* volitional

The only correlation between perceived pain during the NxES and change in PPT was observed in the right knee (Table 3) and the change in PPT at the different sites.

**Table 3:** Relationships between perceived pain VAS and ΔPPT during NMES and NxES

Pairs	Pearsons r, *p* value
NMES VAS vs. ΔPPT R Knee	-.070, *p* = .775
NMES VAS vs. ΔPPT L Knee	.319, *p* = .183
NMES VAS vs. ΔPPT Finger	.164, *p* = .502
NxES VAS vs. ΔPPT R Knee	-.475, *p* = .040*
NxES VAS vs. ΔPPT L Knee	.154, *p* = .528
NxES VAS vs. ΔPPT Finger	-.212, *p* = .384

*VAS* Visual analog scale rating of perceived pain, *NMES* Neuromuscular electrical stimulation, *NxES* Noxious electrical stimulation, Δ*PPT* Change in pressure pain threshold

^*^ indicates statistical significance at the level *p* ≤ .05

**Fig. 4:**
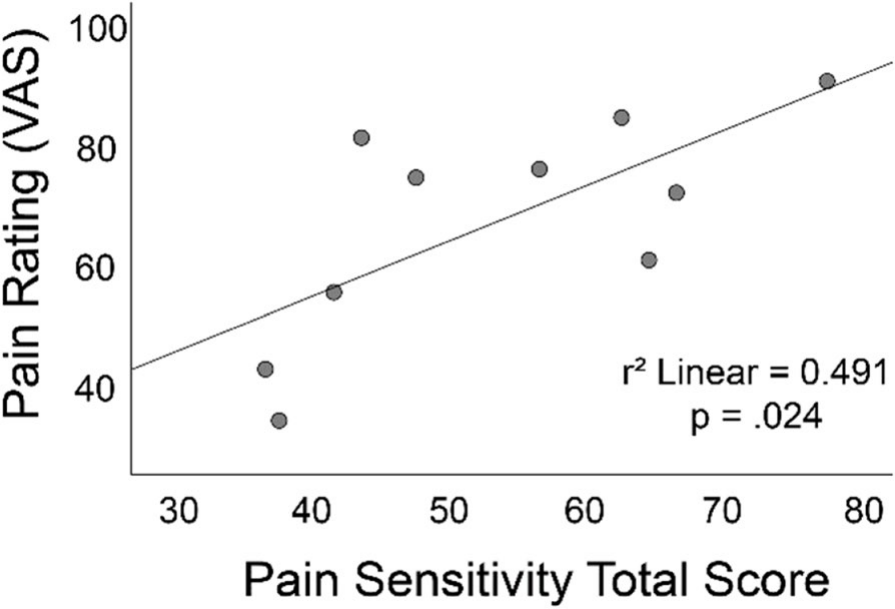
Relationship between the total score on the Pain Sensitivity Questionnaire and pain rating during the noxious stimulation

To assess the relationship between self-reported sensitivity to pain, a subset of participants completed the Pain Sensitivity Questionnaire (PSQ)16 (16). Higher PSQ scores indicate greater sensitivity to pain. A significant relationship was observed between self-reported pain sensitivity and pain perceived during the noxious stimulation treatment. Linear regression analysis demonstrated a significant relationship between pain sensitivity and pain perceived during the noxious stimulation (*r*^2^ = 0.491, *p* = 0.024) (Fig. 4).

## Discussion

The results of this study demonstrate that electrically induced muscle contractions are uncomfortable, though not as uncomfortable as noxious electrical stimulation, and that the discomfort produces a pain inhibitory response. This finding supports our first hypothesis that NMES elicited contractions would produce a pain inhibitory effect no different from NxES. However, our hypothesis that pain inhibition would occur at all three testing sites was not supported; inhibition of pressure pain was not observed in the finger after the painful interventions, which provides insight into the mechanisms involved in this inhibitory pain response.

Pain inhibition is complex and multifactorial and less is known about pain mechanisms in humans compared to animal models. None-the-less, the mechanisms that could be involved in the reduced pressure pain sensitivity observed in this study include exercise induced hypoalgesia (EIH), long term depression (LTD) of nociceptive synaptic connections [23], and descending inhibition via mechanisms involved in diffuse noxious inhibitory control (DNIC)/conditioned pain modulation (CPM). Studies of EIH often involve muscle contractions that are painful [24, 25] so it is plausible that one of the mechanisms involved in EIH is conditioned pain modulation induced by a painful conditioning stimulus produced by painful muscle contractions. However, it is unlikely that EIH is involved in the pain reduction in this study because of the low force and short duration of the contractions used in the volitional or electrically elicited contractions [26]. Inhibition of the transmission of noxious stimuli can result from long term depression of dorsal horn synapses. Long term depression can be induced with either low frequency electrical stimulation [27] or high frequency stimulation produced pain inhibition [23]. Another mechanism that may be involved in pain inhibition when electrical stimulation is applied across the skin is the activation of large diameter Aβ fibers, which was unavoidable in our experimental paradigm. It is well known that input from large diameter Aβ fibers can reduce the transmission of signals from nociceptors to the brain through complex interactions in the substantia gelatinosa of the spinal cord, similar to the original descriptions of the Gate Control Theory [28, 29]. Lastly, the brainstem pain-modulation system may have been involved in pain inhibition. In this system, neurons in the periaqueductal gray area in the midbrain exert an inhibitory effect on dorsal horn neurons indirectly, through projections to the rostral ventral medial medulla, subnucleus reticularis dorsalis and locus coeruleus – a mechanism involving endogenous opioids [30]. Projections from the locus coeruleus, subnucleus reticularis dorsalis, and rostral ventral medial medulla make direct and indirect contact with the nociceptive transmission/tract neurons in the dorsal horn and produce inhibition through endogenous opioids, serotonin, and noradrenalin [30–34].

While this study was not designed to elucidate the exact mechanisms of the inhibitory response, the observation that painful stimuli applied to the right leg, produced pain inhibition in both legs but not in the upper extremity suggests the involvement of spinal level inhibitory mechanisms rather than those exerting more global descending inhibition from supraspinal centers [35, 36]. This is in contrast to research that has demonstrated systemic effects of CPM [25, 26, 37] acting to reduce pain, at least in a healthy sample of participants.

Interestingly, neither the degree of discomfort nor the level of muscle force were found to be related to the amount of pain inhibition experienced by the participants. However, self-reported sensitivity to pain explained almost 50% of the variance in the discomfort experienced during the noxious electrical stimulation. While this finding is not surprising, it indicates that more time may be required to educate patients with greater overall sensitivity to pain in the benefits of electrically elicited muscle strengthening. The importance of patient education should not be underestimated, given that expectation of pain relief from an intervention can affect the outcome of the treatment [38]. Neuromuscular electrical stimulation is commonly used in physical therapy to increase the force generating capacity of a muscle (strength). NMES is particularly useful in people with voluntary muscle activation failure [2, 39] because NMES activates muscle fibers through the motor neurons thereby bypassing the nervous system. The parameters used in NMES and, most importantly, the intensity of electrical stimulation (represented as a percentage of maximum contraction force) are key to success during the use of NMES. Typical doses involve relatively high force contractions (≥ 30–50% MVIC) for 10 s followed by substantial time for the muscle to recover (50–90 s). In contrast, studies of exercise induced hypoalgesia often involve muscle contractions that last much longer than the duration used in the present study [37, 40]. The stimulation parameters and dose used in this study were similar to those recommended for strengthening via electrical stimulation, [41–43] so we could investigate the unintended pain-relieving effect electrically elicited muscle contractions. The results raise important considerations about the effect of muscle strengthening in people with musculoskeletal conditions. Exercise prescribed to increase function often targets muscle strength and endurance, which may help to improve the biomechanical orientation of joints or affect joint stability. However, exercise can also reduce the perception of pain, which is an important consideration when planning treatments for people with strength deficits and pain.

While this study provides evidence for the pain inhibiting influence of NMES elicited contractions, it is not without limitations. The sample size of this study is small, however the fully within-subjects design limits variability between groups and the effect sizes observed were quite strong suggesting robust outcomes. Young healthy individuals were intentionally recruited for the study to avoid impairments in pain processing that can accompany musculoskeletal conditions [20, 44, 45]. However, people with painful conditions due to acute injury (nociception) or nociplastic changes due to chronic pain may respond differently to the intervention. Finally, the intensity of the muscle contractions was lower than what would be prescribed for muscle strengthening. However, had we used 30–50% MVIC contraction intensity, we would expect higher pain ratings that we would expect to lead to an even greater degree of pain inhibition.

## Conclusion

Neuromuscular electrical stimulation is well suited for muscle strengthening due to the unique characteristics of muscle activation at the level of motor axons (increased activation of large, strong, fast-twitch motor units at low levels of force) and due to facilitation of motor unit activation in the central nervous system. While discomfort may be a barrier to the use of NMES, clinicians must fully educate patients to understand the benefits of NMES. This study shows that an *unintended benefit* of NMES is pain inhibition that may increase the acceptability of this beneficial treatment.

## Key points

NMES for strengthening produce strength gains in more large, fast twitch muscle fibers than volitional contractions at the same force level. NMES is considered painful by some, which can limit its use clinically. However, the pain induced during NMES also elicits pain inhibition that could augment the effect of strengthening that could further improved function.

## Data Availability

The datasets used and/or analyzed during the current study are not publicly available because of assurances of privacy that were made to the participants during the consenting process. However, the data are available from the corresponding author on reasonable request.

## Abbreviations

CPM: Conditioned pain modulation
EIH: Exercise induced hypoalgesia
F: Force
MVIC: Maximum voluntary isometric contraction
NMES: Neuromuscular electrical stimulation
NXES: Noxious electrical stimulation
PSQ: Pain sensitivity questionnaire
PPT: Pressure pain thresholds
VAS: Visual analog scale for perceived pain
VOL: Volitional contractions

## References

[1] Rice DA, Graven-Nielsen T, Lewis GN, McNair PJ, Dalbeth N (2015). The effects of experimental knee pain on lower limb corticospinal and motor cortex excitability. Arthritis Res Ther.

[2] Rice DA, McNair PJ, Lewis GN, Dalbeth N (2014). Quadriceps arthrogenic muscle inhibition: the effects of experimental knee joint effusion on motor cortex excitability. Arthritis Res Ther..

[3] Loyd BJ, Burrows K, Forster JE, Stackhouse SK, Hogan C, Stevens-Lapsley JE (2019). Reliability and precision of single frequency bioelectrical impedance assessment of lower extremity swelling following total knee arthroplasty. Physiother Theory Pract..

[4] Stackhouse SK, Eisennagel A, Eisennagel J, Lenker H, Sweitzer BA, McClure PW (2013). Experimental pain inhibits infraspinatus activation during isometric external rotation. J Shoulder Elbow Surg..

[5] Florjanski W, Malysa A, Orzeszek S, Smardz J, Olchowy A, Paradowska-Stolarz A, Wieckiewicz M. Evaluation of biofeedback usefulness in masticatory muscle activity management-a systematic review. J Clin Med. 2019. 10.3390/jcm8060766. 10.3390/jcm8060766 PMC6616888 31151198

[6] Morales-Sanchez V, Falco C, Hernandez-Mendo A, Reigal RE. Efficacy of Electromyographic biofeedback in muscle recovery after meniscectomy in soccer players. Sensors (Basel). 2022. 10.3390/s22114024. 10.3390/s22114024 PMC9185253 35684645

[7] Pietrosimone B, McLeod MM, Florea D, Gribble PA, Tevald MA (2015). Immediate increases in quadriceps corticomotor excitability during an electromyography biofeedback intervention. J Electromyogr Kinesiol..

[8] Stevens JE, Mizner RL, Snyder-Mackler L (2004). Neuromuscular electrical stimulation for quadriceps muscle strengthening after bilateral total knee arthroplasty: a case series. J Orthop Sports Phys Ther..

[9] Snyder-Mackler L, Delitto A, Bailey SL, Stralka SW (1995). Strength of the quadriceps femoris muscle and functional recovery after reconstruction of the anterior cruciate ligament. A prospective, randomized clinical trial of electrical stimulation. J Bone Joint Surg..

[10] Beaumont E, Guevara E, Dubeau S, Lesage F, Nagai M, Popovic M (2014). Functional electrical stimulation post-spinal cord injury improves locomotion and increases afferent input into the central nervous system in rats. J Spinal Cord Med..

[11] Enoka RM, Amiridis IG, Duchateau J (2020). Electrical Stimulation of Muscle: Electrophysiology and Rehabilitation. Physiology (Bethesda)..

[12] Hong IK, Choi JB, Lee JH. Cortical changes after mental imagery training combined with electromyography-triggered electrical stimulation in patients with chronic stroke. Stroke. 2012. 10.1161/STROKEAHA.112.663641. 10.1161/STROKEAHA.112.663641 22798329

[13] Lieber RL (2010). Muscle Structure, Function, and Plasticity The Physiological Basis of Rehabilitation.

[14] Olson CB, Carpenter DO, Henneman E. Orderly recruitment of muscle action potentials. Arch Neurol. 1968. 10.1001/archneur.1968.00480060061008. 10.1001/archneur.1968.00480060061008 5726771

[15] Feiereisen P, Duchateau J, Hainaut K (1997). Motor unit recruitment order during voluntary and electrically induced contractions in the tibialis anterior. Exp Brain Res..

[16] Knaflitz M, Merletti R, De Luca CJ (1990). Inference of motor unit recruitment order in voluntary and electrically elicited contractions. J Appl Physiol..

[17] Glaviano NR, Saliba S (2016). Can the use of neuromuscular electrical stimulation be improved to optimize quadriceps strengthening?. Sports Health..

[18] O’Connor D, Brennan L, Caulfield B. The use of neuromuscular electrical stimulation (NMES) for managing the complications of ageing related to reduced exercise participation. Maturitas. 2018. 10.1016/j.maturitas.2018.04.009. 10.1016/j.maturitas.2018.04.009 29903643

[19] Yarnitsky D, Bouhassira D, Drewes AM, Fillingim RB, Granot M, Hansson P, Landau R, Marchand S, Matre D, Nilsen KB, Stubhaug A, Treede RD, Wilder-Smith O. Recommendations on practice of conditioned pain modulation (CPM) testing. Eur J Pain. 2015. 10.1002/ejp.605. 10.1002/ejp.605 25330039

[20] Arendt-Nielsen L, Nie H, Laursen MB, Laursen BS, Madeleine P, Simonsen OH, Graven-Nielsen T. Sensitization in patients with painful knee osteoarthritis. Pain. 2010. 10.1016/j.pain.2010.04.003. 10.1016/j.pain.2010.04.003 20418016

[21] Ruscheweyh R, Marziniak M, Stumpenhorst F, Reinholz J, Knecht S (2009). Pain sensitivity can be assessed by self-rating: development and validation of the Pain Sensitivity Questionnaire. Pain..

[22] Ruscheweyh R, Verneuer B, Dany K, Marziniak M, Wolowski A, Colak-Ekici R, Schulte TL, Bullmann V, Grewe S, Gralow I, Evers S, Knecht S, Çolak-Ekici R, Schulte TL, Bullmann V, Grewe S, Gralow I, Evers S, Knecht S (2012). Validation of the pain sensitivity questionnaire in chronic pain patients. Pain..

[23] Liu XG, Morton CR, Azkue JJ, Zimmermann M, Sandkuhler J (1998). Long-term depression of C-fibre-evoked spinal field potentials by stimulation of primary afferent A delta-fibres in the adult rat. Eur J Neurosci..

[24] Koltyn KF, Brellenthin AG, Cook DB, Sehgal N, Hillard C. Mechanisms of exercise-induced hypoalgesia. J Pain. 2014. 10.1016/j.jpain.2014.09.006. 10.1016/j.jpain.2014.09.006 PMC4302052 25261342

[25] Koltyn KF, Umeda M. Contralateral attenuation of pain after short-duration submaximal isometric exercise. J Pain. 2007. 10.S1526-5900(07)00737-7 [pii]. 10.1016/j.jpain.2007.06.003 17681886

[26] Kosek E, Ekholm J, Hansson P (1996). Modulation of pressure pain thresholds during and following isometric contraction in patients with fibromyalgia and in healthy controls. Pain..

[27] Jung K, Rottmann S, Ellrich J (2009). Long-term depression of spinal nociception and pain in man: influence of varying stimulation parameters. Eur J Pain..

[28] Braz J, Solorzano C, Wang X, Basbaum AI (2014). Transmitting pain and itch messages: a contemporary view of the spinal cord circuits that generate gate control. Neuron..

[29] Melzack R, Wall PD. Pain mechanisms: a new theory. Science. 1965. https://doi.org/10.1126/science.150.3699.971. 10.1126/science.150.3699.971 5320816

[30] Mills EP, Keay KA, Henderson LA. Brainstem pain-modulation circuitry and its plasticity in neuropathic pain: insights from human brain imaging investigations. Front Pain Res (Lausanne). 2021 https://10.3389/fpain.2021.705345 10.3389/fpain.2021.705345 PMC8915745 35295481

[31] De Resende MA, Silva LFS, Sato K, Arendt-Nielsen L, Sluka KA (2011). Blockade of opioid receptors in the medullary reticularis nucleus dorsalis, but not the rostral ventromedial medulla, prevents analgesia produced by diffuse noxious inhibitory control in rats with muscle inflammation. J Pain..

[32] Wen YR, Wang CC, Yeh GC, Hsu SF, Huang YJ, Li YL, Sun WZ (2010). DNIC-mediated analgesia produced by a supramaximal electrical or a high-dose formalin conditioning stimulus: Roles of opioid and α2-adrenergic receptors. J Biomed Sci..

[33] Khan HS, Stroman PW (2015). Inter-individual differences in pain processing investigated by functional magnetic resonance imaging of the brainstem and spinal cord. Neuroscience..

[34] Bannister K, Dickenson AH (2017). The plasticity of descending controls in pain: translational probing. J Physiol..

[35] Jensen TS (1997). Opioids in the brain: supraspinal mechanisms in pain control. Acta Anaesthesiol Scand.

[36] Sun J, Chen SR, Pan HL. mu-Opioid receptors in primary sensory neurons are involved in supraspinal opioid analgesia. Brain Res. 2020. 10.1016/j.brainres.2019.146623. 10.1016/j.brainres.2019.146623 PMC6946609 31881186

[37] Bement MKH, Dicapo J, Rasiarmos R, Hunter SK, HoegerBement MK, Dicapo J, Rasiarmos R, Hunter SK (2008). Dose response of isometric contractions on pain perception in healthy adults. Med Sci Sports Exerc.

[38] Atlas LYTY, Wager TD. How expectations shape pain. Neurosci Lett. 2012. 10.1016/j.neulet.2012.03.039. 10.1016/j.neulet.2012.03.039 22465136

[39] Rice DA, McNair PJ (2010). Quadriceps arthrogenic muscle inhibition: neural mechanisms and treatment perspectives. Semin Arthritis Rheum.

[40] Naugle KM, Fillingim RB, Riley JL, Riley JL 3rd, Riley JL. A meta-analytic review of the hypoalgesic effects of exercise. J Pain. 2012. 10.1016/j.jpain.2012.09.006. 10.1016/j.jpain.2012.09.006 PMC3578581 23141188

[41] Bade MJ, Stevens-Lapsley J (2011). Early high-intensity rehabilitation following total knee arthroplasty improves outcomes. J Orthop Sports Phys Ther..

[42] Stevens-Lapsley J, Balter JE, Wolfe P, Eckhoff DG, Kohrt WM (2012). Early neuromuscular electrical stimulation to improve quadriceps muscle strength after total knee arthroplasty: a randomized controlled trial. Phys Ther..

[43] Stevens-Lapsley J, Balter JE, Wolfe P, Eckhoff DG, Schwartz RS, Schenkman M, Kohrt WM (2012). Relationship between intensity of quadriceps muscle neuromuscular electrical stimulation and strength recovery after total knee arthroplasty. Phys Ther..

[44] Stackhouse SK, Eckenrode B (2014). Chronic Achilles Tendinopathy Is Associated With Signs of Central Sensitization [abstract]. J Orthop Sports Phys Ther.

[45] Woolf CJ. Central sensitization: Implications for the diagnosis and treatment of pain. Pain. 2011;152. 10.1016/j.pain.2010.09.030. 10.1016/j.pain.2010.09.030 PMC3268359 20961685

